# Psychometric properties of the arabic version of the maslach burnout inventory-human services survey (MBI-HSS) among lebanese dentists

**DOI:** 10.1186/s12903-023-03169-7

**Published:** 2023-07-05

**Authors:** Sanaa Bassam, Heba Mohsen, Zainab Barakat, Linda Abou-Abbas

**Affiliations:** 1 Neuroscience Research Center, Faculty of Medical Sciences, Lebanese University, Beirut, Lebanon; 2 Tropical Neuroepidemiology, Institute of Epidemiology and Tropical Neurology, INSERM, Univ. Limoges, IRD, U1094 GEIST, Limoges, 87000 France; 3 Clinical and Epidemiological Research Laboratory, Doctoral School of Science and Technology, Lebanese University, Beirut, Lebanon; 4 INSPECT-LB (Institut de Sante’ Publique, Epidemiologie Clinique et Toxicologie-Liban), Beirut, Lebanon

**Keywords:** Arabic Version, Burnout, Dentists, Lebanon, Maslach Burnout Inventory-Human Services Survey, Psychometrics

## Abstract

**Background:**

Dentists are at risk of burnout syndrome, which can have negative impacts on their work environment and productivity. Assessing burnout is crucial for maintaining the well-being and effectiveness of dentists in their profession. The present study aims to evaluate the psychometric properties of the Arabic version of the Maslach Burnout Inventory Human Services Survey (MBI-HSS) among dentists.

**Methods:**

The original English version of the MBI-HSS was translated into Arabic, and then back-translated into English by experienced bilingual professionals. Lebanese dentists were asked to participate in the study between February and June 2019. Data collected included demographic information and items from the Arabic version of the MBI-HSS.

**Results:**

A total of 441 people participated in the study, of whom 58.3% were men. The mean age of the sample was 39.6 years (SD = 12.8), with a range of 23 to 68 years old. Approximately 60% of dentists were specialists. Cronbach’s alphas were as follows: emotional exhaustion (alpha = 0.855), depersonalization (alpha = 0.823), and personal achievement (alpha = 0.667). The results of the test-retest reliability assessment demonstrated the strong reproducibility of the MBI-HSS [EE, ICC = 0.927 (0.845, 0.966), p-value < 0.0001; PA, ICC = 0.963 (0.921–0.983), p-value < 0.001; DP, ICC = 0.764 (0.497–0.889), p-value < 0.0001]. The exploratory factor analysis of the MBI-HSS yielded three psychometrically robust sub-domains representing dimensions of “emotional exhaustion,” “depersonalization,” and “personal achievement,” which explained 57.8% of the scale’s total variance. The confirmatory factor analysis revealed that the 15-item model (excluding items 4, 5, 12, 13, 16, 20, and 22) was the most fitting for the data.

**Conclusions:**

The Arabic version of the MBI-HSS scale demonstrated good psychometric properties in Lebanese dentists. However, it would be important to conduct further research to confirm its reliability and validity in other Arab countries.

## Introduction

Research has shown that dentistry is one of the most stressful professions among healthcare workers, with dentists facing a variety of work-related stressors that can negatively impact their physical and mental well-being [1]. These stressors include prolonged working hours, time and scheduling pressures, high noise levels, posture maintenance for long periods, income dissatisfaction, patient demands, and social isolation [2, 3]. Studies have found that the work environment of dentists can greatly influence their health, and that chronic stress can lead to significant psychological issues such as anxiety, depression, and burnout [4].

Burnout refers to gradual depletion of a person manifested by emotional exhaustion, depersonalization, and diminished work efficacy [5]. It is a psychological syndrome that results from prolonged interpersonal stress in workplace [6]. Workers who develop burnout syndrome may show signs of personality change, memory disturbances, and concentration problems [7]. Dentists are particularly susceptible to this condition due to the nature of their work [8, 9], including dealing with anxious patients and managing staff [10, 11]. If left unaddressed, prolonged burnout can lead to serious health problems such as cardiovascular diseases, musculoskeletal problems, and mental illnesses like depression [9, 12–14]. In severe cases, depression could culminate in suicide [15].

Numerous research studies have assessed burnout among dentists worldwide [16–18]. One study conducted in the United States found that one in eight dentists suffers from burnout [19], while another study in Northern Ireland revealed that more than 26% of dental staff are at high risk of burnout [20]. Moreover, a study in the Netherlands found that 21% of participants had a certain level of burnout risk, 13% had high overall levels of burnout, and 2.5% were highly burned out [21]. Burnout is considered a serious risk in dentistry that should be measured frequently among all dentists [22]. Being aware of burnout and managing its symptoms, consequently, improves job satisfaction, patient care, and organizational outcomes [23, 24].

The Maslach Burnout Inventory (MBI) is widely recognized as the “gold standard” for burnout assessment and estimation [25]. There are three main versions of the MBI that measure same burnout dimensions (depersonalization, emotional exhaustion, and personal accomplishment), but are tailored to different occupations. These forms are MBI-General Survey, MBI-Educators Survey, and MBI-Human Services Survey (MBI-HSS) [26].

The MBI-HSS is a commonly used instrument for assessing burnout across three dimensions: emotional exhaustion (EE), depersonalization (DP), and personal accomplishment (PA) [27]. The EE subscale includes nine items that measure feelings of exhaustion and frustration towards work. The DP subscale comprises five items that assess feelings of detachment or cynicism towards patients. Finally, the PA subscale includes eight items that measure a sense of achievement and fulfillment from work. The MBI-HSS has proven to be a reliable and valid instrument for assessing burnout in a variety of human service professions such as dentists [28, 29], nurses [30], and medical personnel [31–33]. The MBI-HSS has been translated into several languages and validated in Italy [30], Spain [34], Serbia [35], the Gulf Cooperation Council Region [32] and Iran [29].

In 2012, a study was conducted in Lebanon to validate the Maslach Burnout Inventory-Human Services Survey (MBI-HSS) among nurses, but the sample used was not representative of the entire Lebanese healthcare professionals, which limits its generalizability. Furthermore, the authors did not adapt the tool to Lebanese culture; instead, they used an existing Arabic version of the scale. Therefore, it is necessary to develop a Lebanese Arabic version of the MBI-HSS specifically for dentists, which could provide more accurate and relevant results for this population. Cross-cultural adaptation is an essential step to ensure linguistic equivalence and comparability of results across different cultures and professions. Validating the MBI-HSS instrument among different health professions can enhance our understanding of burnout and inform the development of evidence-based strategies to promote employee well-being and improve organizational performance. Thus, the objective of this study is to cross-culturally adapt and evaluate the psychometric properties of the MBI-HSS among Lebanese dental professionals.

## Methods

### Study design and sampling procedure

A cross-sectional study was conducted among Lebanese dentists from February to June 2019. Participants were recruited through convenience sampling, and all registered dentists with the Lebanese Dental Association (LDA) who were currently practicing dentistry and able to read and understand Arabic were eligible to participate. Dentists who had taken a holiday for more than one-month preceding data collection were excluded from the study. Convenience sampling was considered a more practical and feasible approach to recruit participants more easily and quickly.

### Sample size calculation

According to statistical guidelines, a respondent-to-item ratio of 10:1 is recommended for factor analysis [36] and a minimum of 200 participants for the confirmatory factor analysis (CFA) [37]. In the present study, this ratio was applied, resulting in a sample size of 220 participants completing a 22-item questionnaire for the factorial analysis. To ensure the necessary number of questionnaires for the study, a license was obtained from the copyright holder (Mind Garden, Inc., USA) to produce 500 hard copies.

### Procedure

Eligible dentists were approached by the researcher, informed about the study’s purpose, and asked to fill out a self-reported anonymous questionnaire. They were also notified, both orally and by written consent, that participation was voluntary. They had the right to refuse or withdraw from the study at any time. After receiving signed informed consent, the questionnaires were distributed to 500 dentists at their workplaces by an investigator. The questionnaires included information on basic sociodemographic and work-related characteristics, as well as the Arabic version of the MBI-HSS. To assess the test-retest reliability of the Arabic version of the MBI-HSS, 30 dentists completed the questionnaire twice, with a 14-day interval between the test and the retest. The study was approved by the scientific committee of the Neuroscience Research Center (NRC) at the Faculty of Medical Sciences at Lebanese University. All necessary measures were taken to protect the anonymity and confidentiality of participants.

## Instrumentation

**The MBI-HSS** is a 22-item instrument that assesses burnout across three dimensions [27]. The emotional exhaustion subscale (EE) includes nine items (I feel emotionally drained from my work, I feel used up at the end of the work day, I feel fatigued when I get up in the morning and have to face another day on the job, I feel like I am at the end of my rope, I feel burned out from my work, I feel frustrated by my job, I feel I’m working too hard on my job, working with people directly puts too much stress on me, working with patients all day is really a strain for me), while the depersonalization subscale (DP) comprises five items (I feel I treat some patients as if they were impersonal objects, I have become more callous toward patients since I took this job, I worry that this job is hardening me emotionally, I don’t really care what happens to some patients, I feel patients blame me for some of their problems), and the personal accomplishment subscale (PA) includes eight items (I can easily understand how my patients feel about things, I deal very effectively with the problems of my patients, I feel I am positively influencing other patients’ lives through my work, In my work, I feel very energetic, I can easily create a relaxed atmosphere with my patients, I feel exhilarated after working closely with my patients, I have accomplished many worthwhile things in the job, I deal with emotional problems very calmly). Each item asks respondents to describe their feelings on a 7-point Likert-type scale, ranging from (0) never having those feelings to [6] having those feelings every day. Higher scores relating to EE and DP correspond to a higher degree of burnout. However, a high score for PA corresponds to a lower degree of burnout in that dimension.

### Translation and cross-cultural adaptation process

To adapt the English version of the MBI-HSS to the Arabic language, we followed the five steps proposed by Beaton et al. [38]. Permission was obtained from Mind Garden, Inc. to translate the original MBI-HSS questionnaire into Arabic. Two certified bilingual translators, whose native language is Arabic, independently translated the MBI-HSS scale from English to Arabic. They were instructed to avoid literal translation and use a simple and understandable language for the Lebanese population. The research team reviewed the two Arabic versions, resolving any inconsistencies by consensus in collaboration with the translators. A joint version was then synthesized. Back translations were then performed by two English speakers who were not familiar with the original English version. The two back-translated versions were compared to the initial English version by a committee of experts consisting of the research team, a translator, and ten dentists. Discrepancies were discussed and resolved among the committee members to confirm semantic, idiomatic, and conceptual equivalencies between the original instrument and the translated version. In a pilot study, a preliminary version of the final scale was developed and tested on 15 Lebanese dentists. They were interviewed to determine if they had difficulty or ambiguity responding to the items. The dentists did not report any issues understanding the scale, so no modifications were made to the scale.

### Statistical analysis

Statistical analysis was performed using the Statistical Package for Social Sciences (SPSS) software version 23.0 (IBM Corp., Armonk, NY, USA) and AMOS. Descriptive statistics were reported as means and standard deviations (SD) for continuous variables and frequency (n) with percentages (%) for categorical variables. Exploratory factor analysis (EFA) and confirmatory factor analysis (CFA) were carried out to investigate the factorial validity. These analyses were conducted separately for the MBI-HSS scale using random split-half samples. EFA was performed on the first random-half sub-sample to explore the factor structure of the MBI-HSS using principal components analysis with varimax rotation. The sampling adequacy was assessed by the Kaiser-Meyer-Olkin (KMO) measure and Bartlett’s test of sphericity. The number of factors retained in the scale was determined based on eigenvalues greater than 1 and a visual inspection of the scree plot. Items were removed if they had low communalities (less than 0.4), or high cross-loadings. CFA was performed through structural equation modeling, with maximum likelihood used to examine the fit of the data to the factor structure of the MBI-HSS instrument. Four CFA models were applied: The first model (M1) was tested on the complete 22-item scale. The remaining models (M2, M3, and M4) were tested on shortened versions of the scale (i.e., 20-item, 17-item, and 15-item versions, respectively) to determine the fit of these models. An adequate model fit was considered when χ^2^ / df ≤ 2.0, CFI > 0.90, GFI/NFI > 0.90, and RMSEA < 0.08 [39]. Cronbach’s alpha was used to evaluate the internal consistency of the scale. A coefficient above 0.7 indicates acceptable internal consistency. Test-retest reliability for the next occasion or appointment interval was evaluated through the intra-class correlation coefficient (ICC; average measure) for the MDAS on 30 patients. ICC values between 0.40 and 0.59 are considered fair, values between 0.60 and 0.74 are good, and values between 0.75 and 1.0 are excellent [40]. All statistical tests were two-sided, and the significance level was set at 0.05.

## Results

### Baseline characteristics of the study participants

Out of 500 questionnaires distributed, 441 were returned, resulting in an overall response rate of 88.2%. Of the total participants, 58.3% were male. The mean age of the sample was 39.6 years (SD = 12.8), with a range of 23 to 68 years old. Approximately 60% of dentists were specialists. There was no significant difference in terms of gender, marital status, or profession between the two samples. However, a significant difference was found in terms of age (Table 1).

**Table 1:** Characteristics of the total participants in the study and the Random Split-Half Samples

	All sample (N = 441)	Split Sample 1 (n = 221)	Split Sample 2 (n = 220)	P-value
**Gender n (%)**				0.59
Male	257 (58.3)	126(57.0)	131(59.5)	
Female	184 (41.7)	95(43.0)	89(40.5)	
**Age Mean (SD)**	39.6 (12.8)	41.4(12.9)	33.5(10.3)	0.002*
**Marital status**				0.08
Single	149(34.3)	64(29.4)	85(39.2)	
Married	268(61.6)	143(65.6)	125(57.6)	
Divorced/widowed	18(4.2)	11(5.0)	7(3.2)	
**Profession**				0.95
General practitioner	177(40.1)	89(40.3)	88(40.0)	
Specialist	264(59.9)	132(59.7)	132(60.0)	

N, n: frequency, %: percentage, SD: standard deviation, *P-value < 0.05 is considered significant

## Psychometric properties of the MBI-HSS

### Reliability of the Arabic version of the MBI-HSS Scale

#### Internal consistency

The internal consistency of the MBI-HSS subscales was calculated using Cronbach’s alpha. The following sub-scale alpha values were obtained: emotional exhaustion (alpha = 0.855), depersonalization (alpha = 0.823), and personal achievement (alpha = 0.667). Deleting any item from the construct did not significantly change the alpha level. The values ranged from 0.75 to 0.79 when an item was deleted at baseline (Table 2).

**Table 2:** Internal Consistency of the MBI-HSS (N = 441)

	Scale Mean if Item Deleted	Scale Variance if Item Deleted	Corrected Item-Total Correlation	Cronbach’s Alpha if Item Deleted
**MBI.1**	44.85	154.96	0.51	0.75
**MBI.2**	44.24	151.04	0.59	0.75
**MBI.3**	44.81	154.47	0.49	0.76
**MBI.6**	44.48	152.96	0.49	0.76
**MBI.7**	42.19	164.09	0.38	0.77
**MBI.8**	44.72	151.55	0.55	0.75
**MBI.9**	42.75	163.53	0.34	0.77
**MBI.10**	45.55	158.64	0.39	0.77
**MBI.11**	45.91	161.43	0.36	0.77
**MBI.14**	44.13	152.02	0.51	0.75
**MBI.15**	46.28	175.38	0.09	0.79
**MBI.17**	42.60	164.66	0.33	0.77
**MBI.18**	42.97	171.53	0.14	0.79
**MBI.19**	42.78	165.50	0.30	0.77
**MBI.21**	43.23	166.18	0.22	0.78

#### Test-retest reliability

Test-retest reliability of the Arabic version of the MBI-HSS was assessed for a group of 30 individuals who completed the MBI-HSS scale twice, two weeks apart, using the intraclass correlation coefficient (ICC). The results of the test-retest reliability assessment demonstrated strong reproducibility of the MBI-HSS [EE, ICC = 0.927 (0.845, 0.966), p-value < 0.0001; PA, ICC = 0.963 (0.921–0.983), p-value < 0.001; DP, ICC = 0.764 (0.497–0.889), p-value < 0.0001].

### Validity of the MBI-HSS

The validity of the MBI-HSS was evaluated using exploratory factor analysis. A total of 22 items from the MBI-HSS scale were included in the analysis. The KMO measure of sampling adequacy of 0.864 and the highly statistically significant values of χ^2^ and df (χ^2^ = 2117.16, df = 231, p-value < 0.0001) showed the appropriateness of conducting factor analysis. All items fit their original dimensions, except for items 13 and 20, which rely heavily on two factors (EE and DP). A total of 5 items that either had low communality (items 4, 5, and 22) or were cross-loaded on two factors (items 13 and 20) were removed. The remaining 17 items were retained for further exploratory factor analysis. Inspection of the scree plot and Eigenvalues suggested a three-factor solution for the scale that explained 57.8% of the total variance. Factor 1, which contained seven items (1, 2, 3, 6, 8, 14, and 16), accounted for 25.4% of the total variance and estimated emotional exhaustion. Factor 2, which consisted of seven items (7, 9, 12, 17, 18, 19, and 21), accounted for 22% and reflected personal achievement. Factor 3, which contained three items (10, 11, and 15), contributed 10.4% and reflected depersonalization. Table 3 displays the factor loadings for each item.

Next, a confirmatory factor analysis was performed to test the dimensionality of the MBI-HSS. Four models were examined:

**Model 1** was the theoretical three-factor structure on the complete 22-item scale as suggested by Maslach et al. [41].

**Model 2** was a three-factor structure of a 20-item version of the scale as suggested by Maslach et al. (removing items 12 and 16) [41].

**Model 3** was a three-factor structure on a shortened version of the scale as suggested by our exploratory factor analysis (removing items 4, 5, 13, 20, and 22).

**Model 4**, a shortened version of the scale, was a slight ad hoc modification of M3 that removed items 12 and 16 as suggested by Maslach et al. [41].

**Fig. 1:**
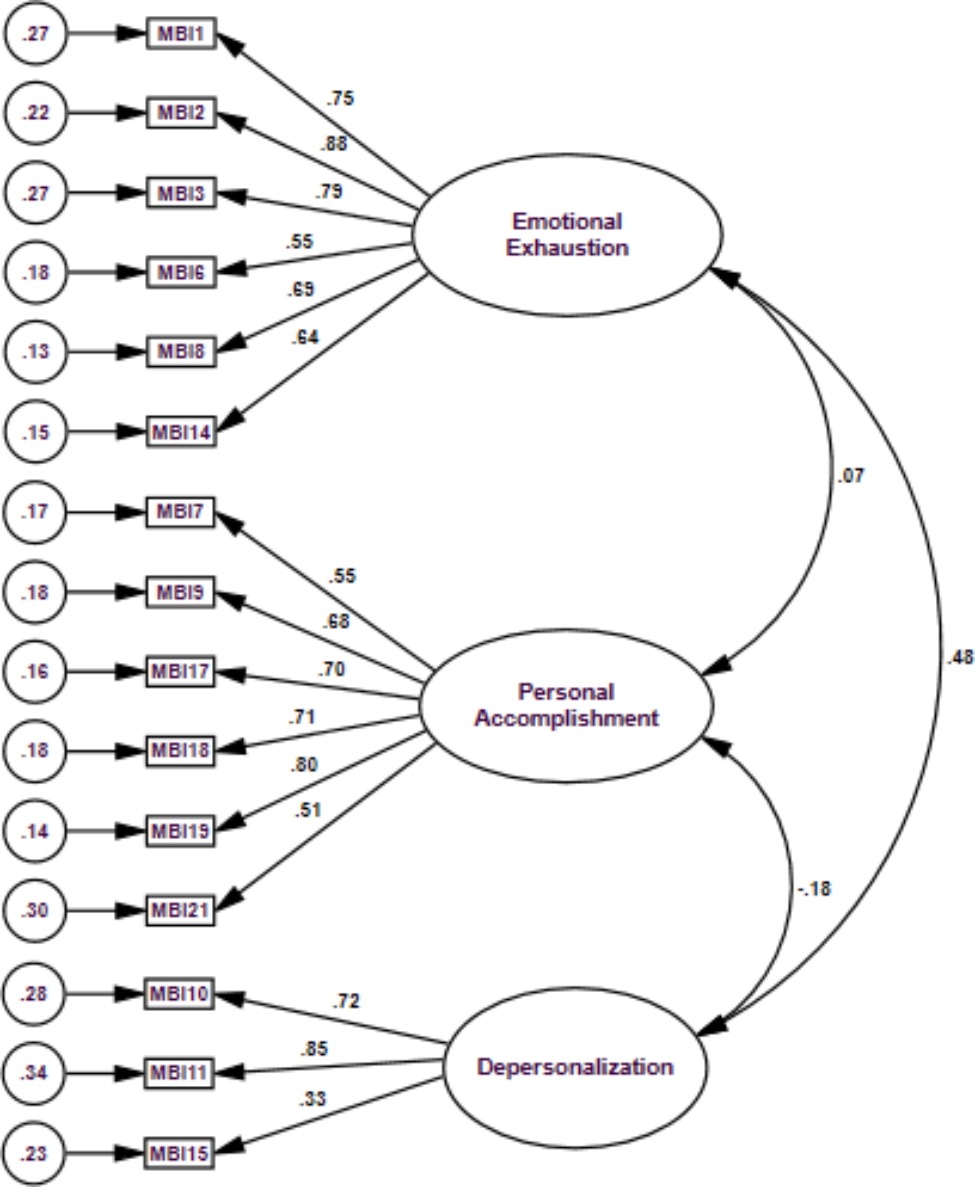
Confirmatory Factor Analysis of the Arabic Version of the MBI-HSS (15 items, excluding items 4, 5, 12, 13, 16, 20, and 22)

Table 4 shows the fit indices for the aforementioned models. The first model displayed a poor fit (χ^2^ = 514.3 (df = 206), which was significant at the P-value ˂ 0.0001, χ^2^/df = 2.5; CFI = 0.83; GFI = 0.83, and RMSEA = 0.08). The second model, which excluded items 12 and 16, did not yield better-fit indices. The third model suggested by the EFA showed somewhat satisfactory performance, as some fit indices reached the anticipated cutoffs (χ^2^/df = 2.0; CFI = 0.91; GFI = 0.88; and RMSEA = 0.07). The fourth model showed substantial improvement in fit indices, with all parameters reaching the desired threshold values (χ^2^/df = 1.8; CFI = 0.91; GFI = 0.94; and RMSEA = 0.06 with an upper bound of 90% CI below the desired value of 0.08). Figure 1 presents the three-factor model of the best-fit model of the MBI-HSS (15 items). EE and DP are positively correlated (r = 0.48, P-value ˂ 0.0001), and DP and PA had a weak negative correlation (r = -0.18, P-value = 0.037). All standardized factor loadings were significant at a p-value < 0.0001 and ranged from 0.33 (item 15) to 0.88 (item 2).

**Table 3:** Exploratory Factor Analysis of the MBI-HSS

Items	Emotional exhaustion	Personal achievement	Depersonalization	Communality
MBI.2	0.840			0.706
MBI.1	0.833			0.697
MBI.3	0.753			0.616
MBI.8	0.722			0.576
MBI.16	0.688			0.547
MBI.6	0.687			0.554
MBI.14	0.528			0.400
MBI.17		0.788		0.636
MBI.19		0.760		0.604
MBI.18		0.733		0.629
MBI.7		0.730		0.554
MBI.9		0.691		0.496
MBI.12		0.671		0.529
MBI.21		0.658		0.455
MBI.10			0.791	0.722
MBI.11			0.784	0.752
MBI.15			0.514	0.300
Eigenvalue	5.91	4.03	1.51	
Percentage of explained variance	25.4	22.0	10.4	

Extraction method: principal component analysis; Rotation method: Varimax with Kaiser Normalization

**Table 4:** Confirmatory factor analysis of the MBI-HSS models (n = 221)

	χ^2*^	χ^2^/df	GFI	CFI	RMSEA	RMSEA (95% CI)
**M1**	514.3	2.5	0.83	0.81	0.08	0.07–0.09
**M2**	429.3	2.6	0.83	0.84	0.08	0.07–0.09
**M3**	237.4	2.0	0.91	0.88	0.07	0.06–0.08
**M4**	157.4	1.8	0.91	0.94	0.06	0.04–0.07

Notes: χ^2^ chi-square, df degree of freedom, CFI Comparative Fit Index, GFI Goodness of Fit Index, RMSEA Root Mean Square Error of Approximation, * all P-values ˂0.0001

M1 three-factor structure of a 22-item scale

M2 three-factor structure of 20-item version (removing items 12 and 16)

M3 three-factor structure on 17 items (removing items 4, 5, 13, 20 &22)

M4 three-factor structure on 15 items (removing items 4, 5, 12, 13, 16, 20 & 22)

## Discussion

The selection of MBI-HSS for this study was based on its strong psychometric properties and its proven usefulness in measuring burnout among healthcare professionals. The MBI-HSS scale is considered the “gold standard” for measuring burnout [5]. The results of the current study showed that the MBI-HSS has appropriate factorial validity and that its three dimensions provide a suitable variance of burnout dimensions among Lebanese dentists. It also provides preliminary evidence that the MBI-HSS scale is a reliable tool for screening burnout among Lebanese dentists.

The results of our study indicated that the internal consistency of all three dimensions of MBI-HSS was adequate, which suggests that the items in each dimension were measuring the same construct consistently. The EE dimension had the highest Cronbach’s alpha in our study, indicating strong internal consistency within this dimension while the PA dimension had the lowest Cronbach’s alpha, indicating some variability in the items within this dimension. These findings are consistent with previous research studies [32]. Additionally, the test-retest reliability of the MBI-HSS scale was evaluated by re-administering the questionnaire to 30 participants after two weeks. The results indicated that the test-retest reliability of the subscales was good to excellent, which suggests that the MBI-HSS scale is stable over time and can produce consistent results. This consistency is important for researchers and practitioners who may use the scale to monitor burnout levels over time or to evaluate the effectiveness of interventions aimed at reducing burnout. Our findings are consistent with other studies that have examined the test-retest reliability of the MBI-HSS scale [31], providing further support for the scale’s reliability.

The exploratory factor analysis of the MBI-HSS yielded three psychometrically robust sub-domains representing dimensions of “emotional exhaustion”, “depersonalization”, and “personal achievement”, which explained 57.8% of the scale’s total variance, similar to other studies [30, 42–44]. This finding supports the three-dimensional factorial structure of the original Maslach model for MBI-HSS [5]. However, in other studies, a five-factor [45] or two-factor model structure was reported [46, 47]. Our findings from CFA were more in line with studies that suggested excluding a few items would increase the fit of the initial three-factor structure (30, 48, 49). As per the original scale, all items were loaded into their original factors except for five (items 4, 5, 13, 20, and 22). In accordance with this finding, items 13 and 22 have been deleted from a study conducted among Lebanese nurses, as they performed poorly [42]. The item sets of (6, 13, 16, and 22), (1, 2, 5, 12, 14 ,and 19), and (6, 13, 14, 15, 16, 20, and 21) have also been deleted as evidence of MBI-HSS validity in studies carried out in Finland, Belgium, and South Korea, respectively [50–52]. Further, earlier research recommended deleting items 12 and 16 to improve the model’s goodness of fit [43, 53–55]. Our CFA results suggested that the 15-item model (excluding items 4, 5, 12, 13, 16, 20, and 22) is the most suitable fit for the data. In contrast to these findings, recent research by Iranian health professionals has shown that all items in each component fit their embedded constructions effectively. There was no deletion required, so the scale retained its three-factor structure with 22 items [31]. One possible reason for the different factor structures reported in previous studies could be cultural differences. Cultural factors such as language, values, and beliefs can influence the way individuals experience and report burnout symptoms. Therefore, it is possible that cultural differences may affect the factor structure of the MBI-HSS.

Our research is notable for being the first to test the validity and reliability of the Arabic version of the MBI-HSS scale among Lebanese dentists. Another highlight is the use of a standard questionnaire which was easy to administer and effective at assessing burnout. Further strengths are the consistency of our findings with other studies and the adequate sample size. However, our study has certain limitations that should be taken into consideration when interpreting the results. Firstly, the use of a convenience sampling method may have introduced selection bias, which limits the generalizability of the findings to the entire population of Lebanese dentists. Future studies should consider using random sampling methods to increase the representativeness of the sample. Secondly, relying solely on self-reported data may have introduced response bias, as participants may have provided socially desirable answers. Thirdly, the absence of external outcome measurements restricted the ability to assess the MBI-HSS-Dentists’ convergent and discriminatory validity.

## Conclusion

The current study provides evidence supporting the validity and reliability of the Arabic version of the MBI-HSS scale in measuring burnout levels among Lebanese dentists. The 15-item, three-factor version of the scale performed better than the original 22-item version, demonstrating adequate internal consistency and test-retest reliability. Further research is needed to determine the clinical validity of the scale and to test its reliability and validity in other Arab countries to establish well-established and reproducible psychometric properties of the scale. Moreover, it is crucial to investigate the determinants of burnout among dental professionals to develop preventive measures, given the potential long-term effects of occupational stress. Neglecting burnout may have adverse consequences on the dentist personally, the quality of work and the professional image in general. Therefore, future research should focus on uncovering the determinants of burnout among Lebanese dentists, using the validated scale.

## Data Availability

The datasets generated and analyzed during the current study are available from the corresponding author upon reasonable request.

## Abbreviations

CFA: confirmatory factor analysis
DP: depersonalization
EFA: exploratory factor analysis
EE: emotional exhaustion
ICC: intra-class correlation coefficient
KMO: Kaiser-Meyer-Olkin
LDA: Lebanese Dental Association
MBI: Maslach Burnout Inventory
MBI-HSS: Maslach Burnout Inventory Human Services Survey
PA: personal accomplishment
SD: standard deviations
